# Separating common from distinctive variation

**DOI:** 10.1186/s12859-016-1037-2

**Published:** 2016-06-06

**Authors:** Frans M. van der Kloet, Patricia Sebastián-León, Ana Conesa, Age K. Smilde, Johan A. Westerhuis

**Affiliations:** Biosystems Data Analysis, Swammerdam Institute for Life Sciences, University of Amsterdam, Science Park 904, 1098 XH Amsterdam, The Netherlands; Computational Genomics Program, Centro de Investigaciones Príncipe Felipe, Valencia, Spain

**Keywords:** Integrated analysis, Multiple data-sets, JIVE, DISCO, O2-PLS

## Abstract

**Background:**

Joint and individual variation explained (JIVE), distinct and common simultaneous component analysis (DISCO) and O2-PLS, a two-block (X-Y) latent variable regression method with an integral OSC filter can all be used for the integrated analysis of multiple data sets and decompose them in three terms: a low(er)-rank approximation capturing common variation across data sets, low(er)-rank approximations for structured variation distinctive for each data set, and residual noise. In this paper these three methods are compared with respect to their mathematical properties and their respective ways of defining common and distinctive variation.

**Results:**

The methods are all applied on simulated data and mRNA and miRNA data-sets from GlioBlastoma Multiform (GBM) brain tumors to examine their overlap and differences. When the common variation is abundant, all methods are able to find the correct solution. With real data however, complexities in the data are treated differently by the three methods.

**Conclusions:**

All three methods have their own approach to estimate common and distinctive variation with their specific strength and weaknesses. Due to their orthogonality properties and their used algorithms their view on the data is slightly different. By assuming orthogonality between common and distinctive, true natural or biological phenomena that may not be orthogonal at all might be misinterpreted.

**Electronic supplementary material:**

The online version of this article (doi:10.1186/s12859-016-1037-2) contains supplementary material, which is available to authorized users.

## Background

To understand and ultimately control any kind of process, albeit biological, chemical or sociological, it is necessary to collect data that functions as a proxy for these processes. Subsequent statistical data analysis on these data should reveal the relevant information to that process. For hypothesis testing such an approach of theory and measuring can be relatively straightforward especially if the analytical instruments are designed specifically for that purpose. In lack of such hypotheses and using generic but readily available analytical instruments, obvious data structures are rarely observed and extensive data analysis and interpretation are necessary (e.g. untargeted analysis [1], data-mining [2]). To make the data-analysis even more complex, the number of observations (*I*) is usually much smaller than the number of variables (*J*) (e.g. transcriptomics data) which prevents the use of classical regression models. Data-analysis and interpretation of the huge number of variables is possible when the number of variables can be summarized in fewer factors or latent variables [3]. For this purpose methods such as factor analysis (FA) [4] or principal component analysis (PCA) [4] were developed.

In functional genomics research it becomes more and more common that multiple platforms are used to explore the variation in samples for a given study. This leads to multiple sets of data with the same objects but different features. Data integration and/or data fusion methods can then be applied to improve the understanding of the differences between the samples. A new group of low level data fusion methods has recently been introduced that are able to separate the variation in all data-sets.

To investigate if the same latent processes underlie the different data-sets, component analysis can be very useful [5]. The construct of latent variables has properties that enable the integrated analysis of multiple data sets with a shared mode (e.g. same objects or variables). With shared variation across multiple data-sets a higher degree of interpretation is achieved and co-relations between variables across the data-sets become (more) apparent. Methods such as generalised SVD (GSVD), latent variable multivariate regression (LVMR), simultaneous component analysis (SCA) and canonical correlation analysis (CCA) have been used successfully in earlier studies [6–9]. Most of these methods or applications of these methods (i.e. CCA) focuses on the common/shared variation across the data-sets only. The interpretation of data however is not only improved by focussing on what is common but likely as important are those parts that are different from each other. These parts could include for example, measurement errors or other process and/or platform specific variations that would be distinctive for each data-set.

**Fig. 1:**
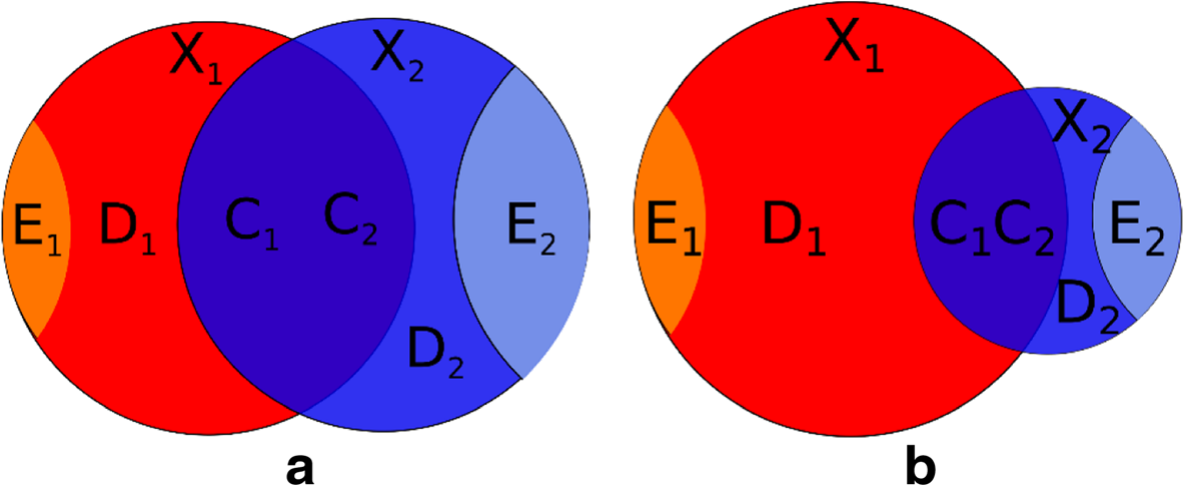
Schematic overview of common and distinctive parts for two data-sets. **a**: two data-sets with equal total variance and **b**: two data-sets with different total variance

The concept of common and distinctive variation is visualized in Fig. 1a and b in which two different situations of overlapping data-sets (**X**_1_(*I* × *J*_1_) and **X**_2_(*I* × *J*_2_)) are shown. The two data-sets are linked via common objects (*I*) but have different variables (*J*_1_ and *J*_2_). The areas of the circles are proportional to the total amount of variation in each data-set. The overlapping parts are tagged as **C**_1_ (*I* × *J*_1_) and **C**_2_ (*I* × *J*_2_) and describe shared (column) spaces for both data-sets. The spaces are not the same but are related (e.g. **C**_1_ = **C**_2_**W**_2 → 1_ + **E**_1_ and **C**_2_ = **C**_1_**W**_1 → 2_ + **E**_2_, in which the **W**'s are the respective weight matrices). Whether or not the residuals **E**_1_ and **E**_2_ are truly zero, depends on the specific method. The distinctive parts **D**_1_ (*I* × *J*_1_) and **D**_2_ (*I* × *J*_2_) describe the variation specific for each data-set and the remainders are indicated by **E**_1_ (*I* × *J*_1_) and **E**_2_ (*I* × *J*_2_). In most methods the common parts are built up from the same latent components.

Figure 1a visualizes **C**_1_ and **C**_2_ as the intersection of the two data-sets. The common parts do not necessarily have to explain a similar amount of variation in each of the sets. The schematic in Fig. 1b demonstrates the situation in which the overlap of the two matrices is proportionally the same for data-set 2 (as in example A) but not for data-set 1.

Attempts have been made to capture both common and distinctive sources of variation across data-sets using GSVD [10], but it has been shown that GSVD does not yield an optimal approximation of the original data in a limited number of components [11]. Alternatives specifically designed for this purpose have been developed and complement the set of low level data fusion methods. In this paper we compare three implementations of such methods (JIVE [12, 13], DISCO-SCA [14, 15] and O2-PLS [16, 17]) with respect to their mathematical properties, interpretability, ease of use and overall performance using simulated and real data-sets. The different approaches to separate common from distinctive variation and the implications on (biological) interpretation are compared. For demonstration purposes we use mRNA and miRNA data from GlioBlastoma Multiform cells available at The Cancer Genome Atlas (TCGA) website [12, 18] as well as simulated data to identify the specific properties of the methods. We will only focus on the integrated analysis of two data-sets that are linked by their common objects. We assume that the data-sets are column-centered. A list of abbreviations and definitions is included in the Appendix.

## Methods

From a general point of view Joint and Individual Variation Explained (JIVE), DIStinct and COmmon simultaneous component analysis (DISCO) and the 2 block latent variable regression with an orthogonal filtering step (O2-PLS) all use a model in which the overlap of two (or more) data-sets is defined as common. The part that is not common is separated into a systematic part called distinctive while the nonsystematic part is called residual. The sum of the common part, the distinctive part and the residual error adds up to the original data-set. The generic decomposition of the two data-sets (**X**_1_ (*I* × *J*_1_) and **X**_2_ (*I* × *J*_2_)) in their respective common and distinctive parts for all three methods can be viewed as:

1 $$ \begin{array}{l}{\mathbf{X}}_1={\mathbf{C}}_1+{\mathbf{D}}_1+{\mathbf{E}}_1\hfill \\ {}{\mathbf{X}}_2={\mathbf{C}}_2+{\mathbf{D}}_2+{\mathbf{E}}_2\hfill \end{array} $$

In which **C**_1_(*I* × *J*_1_) and **C**_2_(*I* × *J*_2_) refer to the common parts, **D**_1_(*I* × *J*_1_) and **D**_2_(*I* × *J*_2_) to the distinctive parts and **E**_1_(*I* × *J*_1_) and **E**_2_(*I* × *J*_2_) to the residual error for both data-sets.

In their respective papers [10, 11, 14] the various authors use different terms that seem to have similar meaning like distinctive, systemic and individual, common and joint etc. For clarity purposes throughout this document we use **common** for combined or joint variation across data sets and **distinctive** for variation specific to each data set. Because the decomposition itself is different for each method, the interpretation of what is common and what is distinctive however, should be placed in the context of the method that is used. We will address the aspects of the different methods in terms of approximations of real data, orthogonalities, explained variance and we will discuss the complexity of proper model selection.

### Algorithms

To compare the three different algorithms it is useful to first briefly reiterate through the different key steps of each method. For the specific implementation the reader is referred to the original papers but for convenience the algorithms are included in the Appendix. The Matlab [19] source code is available for download. Throughout this document the objects (*i* = 1.. *I*) are the rows of the matrices (*I* × *J*) and the variables correspond to the columns (*j* = 1.. *J*). A full list of used symbols and dimensions of the different matrices can be found in the Appendix.

### DISCO

After concatenation of the two matrices, **X**(*I* × *J*) = [**X**_1_(*I* × *J*_1_)|**X**_2_(*I* × *J*_2_)], with *J* = *J*_1_ + *J*_2_), DISCO starts with an SCA routine on the concatenated matrix **X**. This is followed by an orthogonal rotation step of the SCA scores and loadings towards an optimal user-defined target loading matrix P* (i.e. a matrix in which each component is either distinctive for a specific data-set or common for any data-set). As an example, for two data-sets, **X**_1_ (*I* × 2) and **X**_2_ (*I* × 3), with one common component (*c*_*c*_ = 1) and one distinctive component for each data-set (*c*_1_ = *c*_2_ = 1), the total number of components *c*_*t*_ for the whole model is 3.

$$ \mathbf{X}=\left[{\mathbf{X}}_1\Big|{\mathbf{X}}_2\right] $$

$$ \mathbf{X}={\mathbf{U}}_{\left({\boldsymbol{c}}_{\boldsymbol{t}}\right)}{\mathbf{S}}_{\left({\boldsymbol{c}}_{\boldsymbol{t}}\right)}{V}_{\left({\boldsymbol{c}}_{\boldsymbol{t}}\right)}^t $$

$$ {\mathbf{T}}_{sca}={\mathbf{U}}_{\left({\boldsymbol{c}}_{\boldsymbol{t}}\right)} $$

$$ {\mathbf{P}}_{sca}={\mathbf{V}}_{\left({\boldsymbol{c}}_{\boldsymbol{t}}\right)}{\mathbf{S}}_{\left({\boldsymbol{c}}_{\boldsymbol{t}}\right)} $$

$$ \hat{\mathbf{X}}={\mathbf{T}}_{sca}{\mathbf{P}}_{sca}^{\mathrm{t}} $$

And P* is:

$$ {\mathbf{P}}^{*}=\left[\begin{array}{lll}1\hfill & 0\hfill & 1\hfill \\ {}1\hfill & 0\hfill & 1\hfill \\ {}0\hfill & 1\hfill & 1\hfill \\ {}0\hfill & 1\hfill & 1\hfill \\ {}0\hfill & 1\hfill & 1\hfill \end{array}\right] $$

In P*, the zeros are a hard constraint while the ones are not restricted and can be any value. The first two rows relate to the (two) variables in the first data-set, the last 3 rows relate to the variables for the second data-set. The first column relates to the first distinctive component (for data-set 1). The second column is reserved for the distinctive component for the second data-set and the third column is the loading for the common component in both data-sets. Through orthogonal rotation the best rotation matrix (**B**_***opt***_ (*c*_*t*_ × *c*_*t*_)) to rotate the **P**_sca_ loadings (**P**_*r*_) towards the target loadings P* is found by minimizing the squared sum of the 0 entries in the **P**_*r*_ matrix. To do just that a weight matrix (**W** = 1 − P*) is used, in which all the 1 entries are set to 0 and the 0 entries to 1:

$$ {\mathbf{B}}_{opt}\ \underset{min}{\to }\ {\displaystyle \sum }{\left(\mathbf{W}\circ \left({\mathbf{P}}_{\mathrm{sca}}\mathbf{B}\right)\right)}^2 $$ s.t. **B**^**t**^**B** = **I**

**B**_***opt***_ is used to calculate the final rotated scores and loadings (**T**_*r*_ = **T**_*sca*_**B**_*opt*_ and **P**_*r*_ = **P**_*sca*_**B**_***opt***_). Consequently the smallest distance criterion is based only on the 0 entries (in P*) and thus on the distinctive components only. A perfect separation of the distinctive components is often not achieved; the positions where P* is 0 are not exactly 0 in **P**_***r***_. Furthermore, the common variation is forced to be orthogonal to these distinctive parts which clearly could lead to sub-optimal estimations of this common variation. The effects of the orthogonality constraints are discussed later. The final decomposition of the DISCO algorithm is:

2 $$ \begin{array}{l}{\mathbf{X}}_1={\mathbf{C}}_1+{\mathbf{D}}_1+{\mathbf{E}}_1={\mathbf{T}}_c{\mathbf{P}}_{c_1}^{\mathrm{t}}+{\mathbf{T}}_{d_1}{\mathbf{P}}_{d_1}^{\mathrm{t}}+{\mathbf{E}}_1\hfill \\ {}{\mathbf{X}}_2={\mathbf{C}}_2+{\mathbf{D}}_2+{\mathbf{E}}_2={\mathbf{T}}_c{\mathbf{P}}_{c_2}^{\mathrm{t}}+{\mathbf{T}}_{d_2}{\mathbf{P}}_{d_2}^{\mathrm{t}}+{\mathbf{E}}_2\hfill \end{array} $$

The common scores (**T**_*c*_) for both data-sets are the same and are obtained by optimizing on the distinctive components.

### JIVE

The JIVE algorithm is also based on an SCA of the concatenated data-sets (**X**). The common parts for both data-sets (**C**_**k**_) are estimated simultaneously, **C** = [**C**_1_|**C**_2_] = **T**_*sca*_**P**_*sca*_^t^ (*I* × *J*), but now with only the number of common components (*c*_*c*_) and not all the components (*c*_*t*_) like in DISCO. The distinctive parts (**D**_1_ and **D**_2_) are estimated separately and iteratively based on an orthogonal residual (**R**_**k**_ − **T**_*sca*_**T**_*sca*_^t^**R**_*k*_) matrix with *c*_*k*_ distinctive components. Using the same example as before;

$$ \mathbf{X}={\mathbf{U}}_{\left({c}_c\right)}{\mathbf{S}}_{\left({c}_c\right)}{\mathbf{V}}_{\left({c}_c\right)}^t $$

$$ {\mathbf{T}}_{sca}={\mathbf{U}}_{\left({c}_c\right)} $$

$$ {\mathbf{P}}_{sca}={\mathbf{V}}_{\left({c}_c\right)}{\mathbf{S}}_{\left({c}_c\right)} $$

$$ {\mathbf{C}}_{\mathbf{k}}={\mathbf{T}}_{sca}{\mathbf{P}}_{sca}^{\mathrm{t}} $$

$$ {\mathbf{R}}_{\mathbf{k}}={\mathbf{X}}_{\mathbf{k}}-{\mathbf{C}}_{\mathbf{k}} $$

$$ {\mathbf{R}}_{\mathbf{k}}-{\mathbf{T}}_{sca}{\mathbf{T}}_{sca}^{\mathrm{t}}{\mathbf{R}}_k = {\mathbf{U}}_{{\boldsymbol{d}}_{\boldsymbol{k}}\left({\boldsymbol{c}}_{\boldsymbol{k}}\right)}{\mathbf{S}}_{{\boldsymbol{d}}_{\boldsymbol{k}}\left({\boldsymbol{c}}_{\boldsymbol{k}}\right)}{\mathbf{V}}_{{\boldsymbol{d}}_{\boldsymbol{k}}\left({\boldsymbol{c}}_{\boldsymbol{k}}\right)}^{\boldsymbol{t}} $$

$$ {\mathbf{D}}_{\mathbf{k}}={\mathbf{U}}_{d_k\left({\boldsymbol{c}}_{\boldsymbol{k}}\right)}{\mathbf{S}}_{d_k\left({\boldsymbol{c}}_{\boldsymbol{k}}\right)}{\mathbf{V}}_{d_k\left({\boldsymbol{c}}_{\boldsymbol{k}}\right)}^{\mathrm{t}} $$

$$ \mathbf{X}=\mathbf{X}-\left[{\mathbf{D}}_1\Big|{\mathbf{D}}_2\right] $$

The steps are repeated until convergence of the combined common and distinctive matrices (**C** + **D)**. By using the iterative and alternate optimization of the common and distinctinve parts, the orthogonality between the two distinctive parts that does exist in DISCO is no longer enforced. The resulting fit should be able to accommodate more types of data (e.g. the data has to conform to less criteria) than DISCO. Similar to DISCO the common parts are estimated from an SCA on both data-sets simultaneously and like DISCO there is no guarantee that both blocks take part in the common loadings **P**_*sca*_. As a consequence, the optimal solution could for example be one where **P**_*sca*_(=[**P**_1_|**P**_2_]) only has values for **P**_1_ and not **P**_2_ which hardly can be considered common.

The resulting decompostion (Eq. 3) in scores and loadings is exactly the same as for DISCO:

3 $$ \begin{array}{l}{\mathbf{X}}_1={\mathbf{C}}_1+{\mathbf{D}}_1+{\mathbf{E}}_1={\mathbf{T}}_c{\mathbf{P}}_{c_1}^{\mathrm{t}}+{\mathbf{T}}_{d_1}{\mathbf{P}}_{d_1}^{\mathrm{t}}+{\mathbf{E}}_1\hfill \\ {}{\mathbf{X}}_2={\mathbf{C}}_2+{\mathbf{D}}_2+{\mathbf{E}}_2={\mathbf{T}}_c{\mathbf{P}}_{c_2}^{\mathrm{t}}+{\mathbf{T}}_{d_2}{\mathbf{P}}_{d_2}^{\mathrm{t}}+{\mathbf{E}}_2\hfill \end{array} $$

The common scores (**T**_*c*_) for both data-sets are the same. Because SCA is a least squares method and the common parts are determined first, those variables with much variance are likely to end up in the common parts. Because JIVE is an iterative solution the initial guesses for common and distinctive parts can change considerably during these iterations (see Additional file 1). If however, the distinctive variation is larger than the (combined) common variation these iterations will not prevent the method to mis-identify the common components.

### O2-PLS

In contrast to DISCO and JIVE, that use an SCA on the concatenated data-sets, O2-PLS starts with an SVD on the covariance matrix (**X**_1_^t^**X**_2_ (*J*_1_ × *J*_2_)) for an analysis of the common variation. Similar to JIVE, the common components are estimated first and from the orthogonal remainder to ${\mathbf{P}}_{c_k}$ (${\mathbf{R}}_{\boldsymbol{k}}^{\mathrm{t}}{\mathbf{T}}_{c_k}$), per data-set. The distinctive component is estimated per component. When all distinctive components are removed from the data the common scores are updated. Using the same matrices **X**_1_ and **X**_2_;

$$ {\mathbf{X}}_2^{\mathrm{t}}{\mathbf{X}}_1={\mathbf{P}}_{c_1\left({c}_{\mathrm{c}}\right)}{\mathbf{D}}_{\left({c}_{\mathrm{c}}\right)}{\mathbf{P}}_{c_2\left({c}_{\mathrm{c}}\right)}^{\boldsymbol{t}} $$

Deflate **X**_*k*_ per component:

$$ {\mathbf{T}}_{c_k}={\mathbf{X}}_k{\mathbf{P}}_{c_k} $$

$$ {\mathbf{R}}_k={\mathbf{X}}_k-{\mathbf{T}}_{c_k}{\mathbf{P}}_{c_k}^{\mathrm{t}} $$

$$ {\mathbf{R}}_k^{\mathrm{t}}{\mathbf{T}}_{c_k} = {\mathbf{u}}_{d_k(1)}{\mathbf{s}}_{{\boldsymbol{d}}_{\boldsymbol{k}}(1)}{\mathbf{v}}_{d_k(1)}^{\boldsymbol{t}} $$

$$ {\mathbf{t}}_{d_{k,l}}={\mathbf{X}}_k{\mathbf{u}}_{d_k} $$

$$ {\mathbf{p}}_{d_{k,l}}={\left({\mathbf{t}}_{d_{k,l}}^{\mathrm{t}}{\mathbf{t}}_{d_{k,l}}\right)}^{-1}{{\mathbf{X}}_k}^{\mathrm{t}}{\mathbf{t}}_{d_{k,l}} $$

$$ {\mathbf{X}}_k={\mathbf{X}}_k-{\mathbf{t}}_{d_{k,l}}{\mathbf{p}}_{d_{k,l}}^{\mathrm{t}} $$

The choice of a covariance matrix seems appropriate since we are interested in co-varying variables across the data-sets. In case of orthogonal blocks where no common variation exists, the covariation matrix would be 0 and no common variation can be estimated. Similar to JIVE, the distinctive parts are calculated orthogonal to the common part for every data-set individually. Because the common parts are estimates from the individual blocks (not the concatenation) the algorithm itself is less restrictive than JIVE. With different common scores per data-set the decomposition of Eq. 1 in scores and loadings is almost similar to Eqs. 2 and 3;

4 $$ \begin{array}{l}{\mathbf{X}}_1={\mathbf{C}}_1+{\mathbf{D}}_1+{\mathbf{E}}_1={\mathbf{T}}_{c_1}{\mathbf{P}}_{c_1}^{\mathrm{t}}+{\mathbf{T}}_{d_1}{\mathbf{P}}_{d_1}^{\mathrm{t}}+{\mathbf{E}}_1\hfill \\ {}{\mathbf{X}}_2={\mathbf{C}}_2+{\mathbf{D}}_2+{\mathbf{E}}_2={\mathbf{T}}_{c_2}{\mathbf{P}}_{c_2}^{\mathrm{t}}+{\mathbf{T}}_{d_2}{\mathbf{P}}_{d_2}^{\mathrm{t}}+{\mathbf{E}}_2\hfill \end{array} $$

As a post-processing step the common scores can be combined and by means of a regression model [20], for example an SCA of the combined common parts, global common scores can be calculated (i.e. **T**_*c*_ invariant for a block) so Eq. 4 would be exactly Eqs. 2 and 3 [21]. This would however also require recalculation of ${\mathbf{P}}_{c_1}$ and ${\mathbf{P}}_{c_2}$.

### Orthogonalities

The similarity between the three methods is large in terms of scores and loadings that are created in accordance with the algorithms. The methods however are different in terms of constraints that are applied during the decompositions which leads to different orthogonality properties and consequently different independence of the different common and distinctive parts.

The similarity between DISCO and JIVE is a consequence of the use of SCA in both methods. Because the final step in DISCO involves an orthogonal rotation of scores and loadings, the orthogonality between all the rotated scores and loadings remains. This rotation also forces orthogonality between the separate terms: **C**_1_**D**_1_^*t*^ = 0, **C**_1_**D**_2_^*t*^ = 0, **D**_1_**D**_2_^*t*^ = 0, **C**_2_**D**_1_^*t*^ = 0 and **C**_2_**D**_2_^*t*^ = 0. The error terms (**E**_1_ and **E**_2_) are orthogonal to each respective common part and distinctive part only. Orthogonality between the distinctive and common part per data-set in JIVE is enforced by estimation of the distinct components orthogonally to the common scores (${\mathbf{T}}_{sca}\ \left(\mathbf{I}-{\mathbf{T}}_{sca}{\mathbf{T}}_{sca}^t\right){\mathbf{R}}_k={\mathbf{U}}_{{\boldsymbol{d}}_{\boldsymbol{k}}\left({\boldsymbol{c}}_{\boldsymbol{k}}\right)}{\mathbf{S}}_{{\boldsymbol{d}}_{\boldsymbol{k}}\left({\boldsymbol{c}}_{\boldsymbol{k}}\right)}{\mathbf{V}}_{{\boldsymbol{d}}_{\boldsymbol{k}}\left({\boldsymbol{c}}_{\boldsymbol{k}}\right)}^{\boldsymbol{t}}\Big)$. There is no restriction for orthogonality between the distinctive parts of the different data-sets. Because the distinctive parts are calculated as the final step, the error matrix (**E**_*k*_) is orthogonal to the distinctive part but not to the common part.

The decomposition in scores and loadings using the O2-PLS algorithm (Eq. 4) is similar to those obtained when using JIVE or DISCO (Eqs. 2 and 3). The significant difference in terms of orthogonality follows from the fact that there is room for the common parts (i.e. **C**_1_ and **C**_2_) to have different loadings **and** scores. The common scores for each block (${\mathbf{T}}_{c_1}$ and ${\mathbf{T}}_{c_2}$) themselves are expected to have a high correlation because the SVD was applied on the covariance matrix of the two matrices. The distinctive parts are estimated under the restriction that they are orthogonal to the common part per data-set. As a consequence the common parts per data-set share no variance with the distinctive parts. The distinctive parts themselves are not orthogonal to the common parts of the other data-set although the correlations are very small. Similar to JIVE the residuals (**E**_1_ and **E**_2_) in O2-PLS are found to be orthogonal only to the distinctive parts that are calculated as a final step.

A summary of the different orthogonality constraints for the three algorithms can be found in Table 1. It is clear that DISCO is the most strict and O2-PLS the most lenient regarding orthogonality properties. The different constraints that each algorithm imposes will affect the decomposition in different scores and loadings. What is designated as common and what is distinctive per method depends on these constraints. In DISCO the common part is defined as what is orthogonal to the distinctive parts while in JIVE this is the reverse i.e., what is distinctive is what is orthogonal to what is common. From a semantical point of view this seems equivalent but mathematically can generate very different results. These constraints will therefore be of importance when interpreting the data and consequently also for the application of the method. Orthogonality properties make it easier to come to a clear definition of these terms. Furthermore, orthogonality properties make the estimation of the separate parts easier.

**Table 1:** Summary table of all orthogonalities constraints for the three algorithms

	DISCO	JIVE	O2-PLS
Orthogonalities (*k* ≠ *l*)			
***C***_*k*_^t^ ***D***_*k*_	0	0	0
***E***_*k*_^t^ ***C***_*k*_	0	≠0	≠0
***E***_*k*_^t^ ***D***_*k*_	0	0	0
***C***_*k*_^t^ ***D***_*l*_	0	0	≠0
***D***_*k*_^t^ ***D***_*l*_	0	≠0	≠0
***E***_*k*_^t^ ***C***_*l*_	0	≠0	≠0
***E***_*k*_^t^ ***D***_*l*_	0	≠0	≠0
Characteristics			
Fusion	[**X**_1_|**X**_2_]	[**X**_1_|**X**_2_]	**X**_2_^***t***^ **X**_1_
First step	Distinctive	Common	Common
Optimization	Distinctive	Common + Distinctive	Common/Distinctive

(0: orthogonal, ≠0: no forced orthogonality)

The orthogonality constraints between allmost all parts in DISCO enforce that all underlying sources of variation can be split up in orthogonal parts, even the distinctive parts. From a mathematical viewpont this is a perfect separation but in biological phenomena such behavior will be rare. The solution therefore might be easier to find but it makes the interpretation more difficult. In JIVE the orthogonality constraint between the distinctive parts is removed and consequently is expected to be better suitable for biological data. With the single restriction of the distinctive parts to be orthogonal to the common part, O2-PLS is expected to suit most data-sets. The flexibility of O2-PLS is advantageous for fitting the best common and distinctive parts but might come at the expense of more loosely coupled common parts. Furthermore, the distinctive parts in O2-PLS are referred to as orthogonal to the counter common parts (e.g. ***C***_*k*_^t^***D***_*l*_ = 0) and therefore do not optimally describe the total variation in the residual block (**R**_*k*_) which would limit the interpretation of these distinctive parts. The fact that we did not fully observe ***C***_*k*_^t^***D***_*l*_ = 0 but still find some small residuals originates from the updated scores (${\mathbf{T}}_{c_k}={\mathbf{X}}_k{\mathbf{P}}_{c_k}$) after deflation in the algorithm.

### Explained variances

The orthogonalities discussed above imply, because of the centering, uncorrelated structure between the distinctive and common parts. A closer look at the algorithms reveals an additional layer of complexity. This is especially true for DISCO and JIVE where the SVD is taken from the concatenated matrix **X**. The simultaneous decomposition in DISCO:

$$ \mathbf{X}=\left[{\mathbf{X}}_1\Big|{\mathbf{X}}_2\right] $$

$$ \widehat{\mathbf{X}}=\mathbf{T}{\mathbf{P}}^{\mathrm{t}}=\mathbf{T}\mathbf{B}{\mathbf{B}}^{\mathrm{t}}{\mathbf{P}}^{\mathrm{t}}\left({\mathbf{T}}_{rot}=\mathbf{T}\mathbf{B},\ {\mathbf{P}}_{rot}=\mathbf{PB}\right) $$

$$ \widehat{\mathbf{X}}={\mathbf{T}}_{rot}{\mathbf{P}}_{rot}^{\mathrm{t}} $$

$$ \left[{\mathbf{X}}_1\Big|{\mathbf{X}}_2\right]={\mathbf{T}}_{rot}{\mathbf{P}}_{rot}^{\mathrm{t}}+\left[{\mathbf{E}}_1\Big|{\mathbf{E}}_2\right] $$

$$ \left[{\mathbf{X}}_1\Big|{\mathbf{X}}_2\right]=\left[{\mathbf{C}}_1\Big|{\mathbf{C}}_2\right]+\left[{\mathbf{D}}_1\Big|{\mathbf{D}}_2\right]+\left[{\mathbf{E}}_1\Big|{\mathbf{E}}_2\right]=\mathbf{C}+\mathbf{D}+\mathbf{E} $$

decomposes the concatenated data-sets together in orthogonal **combined** parts. The explained variances of the separate parts of the **combined** model add up:

5 $$ \parallel \mathbf{X}{\parallel}^2=\parallel \mathbf{C}{\parallel}^2+\parallel \mathbf{D}{\parallel}^2+\parallel \mathbf{E}{\parallel}^2=\parallel \mathbf{C} + \mathbf{D}+\mathbf{E}{\parallel}^2 $$

∥ **E** ∥ ^2^ is minimal for a given total number of components (*c*_*t*_). The best **P**_*rot*_ however, is an approximation of P* and because of orthogonality constraints, situations can occur where the rotation is not perfect. In such cases the elements set to zero in the original target matrix are different from zero in **P**_*rot*_. The exact estimation of **X**_*k*_ is:

6 $$ {\mathbf{X}}_{\boldsymbol{k}}={\mathbf{T}}_c{\mathbf{P}}_{c_k}^{\mathrm{t}} + {\mathbf{T}}_{d_k}{\mathbf{P}}_{d_k}^{\mathrm{t}}+{\mathbf{T}}_{d_{\ne k}}{\mathbf{P}}_{d_k}^{\mathrm{t}}+{\mathbf{E}}_k $$

The cross-over (${\mathbf{T}}_{d_{\ne k}}{\mathbf{P}}_{d_k}^{\mathrm{t}}\Big)$ part of the original **X**_*k*_, the variation in **X**_*k*_ that is explained by the distinctive components of the other data sets, is minimized during the DISCO iterations and is indicative for the influence both data-sets have on each others individual loadings and thus affect direct interpretation. The size of the cross-over part depends on the data and the number of distinctive components reserved for the other data-sets. The model selection procedure is based on minimization of this cross-over content.

Contrary to DISCO, not all parts in both JIVE and O2-PLS are orthogonal (see Table 1). Equation 5 does not hold and should be reduced, per data-set, to:

7 $$ \parallel {\mathbf{C}}_{\boldsymbol{k}}{\parallel}^2 + \parallel {\mathbf{D}}_{\boldsymbol{k}}{\parallel}^2 = \parallel {\mathbf{C}}_{\boldsymbol{k}} + {\mathbf{D}}_{\boldsymbol{k}}{\parallel}^2 $$

The residual **E**_***k***_ is not orthogonal to the common part **C**_***k***_ which indicates that the final solution found for **E**_***k***_ could still hold some information from **C**_***k***_. To find the correct value for **E**_***k***_ type III partial explained sum of squares for residuals should be applied by projecting **E**_***k***_ on **C**_***k***_ and only consider orthogonal parts of residual [22].

### Interpretation

Even though the fusion methods have separated common from distinctive variation the interpretation of the results can be hampered or sometimes even prohibited by the fact that the data-sets themselves do not conform to the appropriate criteria. The most apparent critereon is the link between the samples across the different data-sets. If the different data-sets for example contain technical replicates, the fusion can only be performed on the averages of the technical replicates as the technical replicates of different data sets are not directly related. Secondly, in order to give equal chance to all data sets to be represented in the model, large blocks should not be favoured just because of their size. Therefore after variable scaling, a block scaling is usually applied such that the sum of squares of all blocks is equal. This block scaling however lowers the influence of the individual variables if the data-set consists of many variables and thus could be the cause of under-estimation.

Common variation can be thought of as variation that is related between data-sets. Because there is no mandatory contribution of both data-sets to the common parts when using JIVE or DISCO the results should always be validated for a shared variation between the data-sets. Second, for blocks where *I* is larger than *J*_*k*_ the rank of data-set **X**_*k*_ is bounded by the number of variables. The selection of the common score **T**_*c*_ from the concatenated matrix **X** defines a direction in the *I*^th^dimensional columnspace that may be outside the *J*_*k*_dimensional subspace in *R*^I^ defined by **X**_*k*_. **C**_*k*_, which is built from **T**_*c*_ will therefore also be outside the *J*_k_ dimensional subspace defined by **X**_*k*_. Thus there will be variation in **C**_*k*_ which is not in **X**_*k*_. When scores ${\mathbf{T}}_{{\boldsymbol{d}}_{\boldsymbol{k}}}$ for the distinctive part **D**_*k*_ are calculated, they are forced to be orthogonal to **T**_*c*_, but not forced to be in the columnspace of **X**_*k*_. This means that also the distinctive part **D**_*k*_ may not be in the columnspace of **X**_*k*_. Because of this, the interpretation of the loadings from **C**_*k*_ and **D**_*k*_ can go wrong, as they may represent variation that is not in **X**_*k*_.

To check whether the distinctive and common parts are still in the column space of the original matrix of the separate data-sets, the projections of **C**_*k*_ and **D**_*k*_ on **X**_*k*_ can be determined via:

8 $$ {\widehat{\mathbf{C}}}_k={\mathbf{X}}_k{\mathbf{X}}_k^{+}{\mathbf{C}}_k $$

The residual (i.e. ∥ **C**_*k*_ − **Ĉ**_*k*_ ∥ ^2^ or $\parallel {\mathbf{D}}_k-{\widehat{\mathbf{D}}}_k{\parallel}^2$) is zero for a perfect projection and different from zero if **C**_*k*_ or **D**_*k*_ is not within the column space of **X**_*k*_.

The common and distinct parts of O2-PLS are based on an SVD of the covariance matrix of **X**_1_ and **X**_2_ ($\left[{\mathbf{P}}_{c_1}\mathbf{D}{\mathbf{P}}_{c_2}\right]=svd\Big({\mathbf{X}}_2^{\mathrm{t}}{\boldsymbol{X}}_1,{c}_c$). The SVD decomposes the covariance matrix in orthogonal contributions. ${\mathbf{P}}_{c_1}$ is expressed in terms of variables of **X**_1_ and ${\mathbf{P}}_{c_2}$ in terms of variables of **X**_2_. The subsequent steps in the algorithm only affect the individual blocks. Consequently, no variation from one data-set is introduced into the other and projection issues like in JIVE and DISCO do not occur. If the post-processing step is performed to calculate global common scores, variation from other data-sets is introduced and also in this case the projection errors need to be evaluated.

The issue that the common scores of multiple data sets may not be in the column space of each data set separately, and the problems this brings was already discussed earlier for multiblock PLS models [23, 24]. In the latter paper the common score was called the super score. It was shown that deflation of information from the separate blocks using the super score leads to introduction of variation that was never present in the block. When information which is not present in the data set is subtracted from that dataset, it is actually (negatively) introduced.

### Model selection

Both orthogonalities and explained variances on touch the heart of exactly what is common and what is distinctive. The three methods are all different in this respect. All three methods however, can only decompose the data-sets if the optimal number of common and distinctive components for the final model are known. It is important that the selected model is appropriate for the data-sets that are analysed and each method has its own strategy of selecting the appropriate model.

Model selection in DISCO is a two step process. In the first step the total number of components (*c*_*t*_) is selected based on proportion of variance accounted for by the simultaneous components for each individual data block. The second step finds the “best” performing model from all possible combinations of common (*c*_*c*_) and distinctive components (${c}_{d_k}$) by minimizing the cross-over parts of each data-set.

In JIVE the configuration of the model is based on the analysis of permuted versions of the original matrix. For the common components complete rows of each data-set are permuted. This removes the link between the objects from the different data-sets, but does not remove the correlation structure inside each block. The eigenvalues for a large number of permuted matrices are determined. The number of common components is defined as that number where the eigenvalues of the original matrix (**X**) are (still) larger than the permuted ones (with a certain α). For the distinct components per data set **X**_*k*_, the rows of each variable in that data-set are permuted to disturb the variable object relationship. Again the eigenvalues of the original data set are compared to the eigenvalues of the permuted data sets to find the optimal number of distinct components for each **X**_*k*_. These setting are used as input for a new start of the estimation of the number of components. This process is repeated until convergence of the number of common and distinctive components.

The model selection of O2-PLS as described in the papers [16, 17] is not clear about exactly which procedures to follow. We have adopted the strategy of first selecting the number of common components based on the covariance matrix followed by an estimation of the number of individual components per data-set using PCA cross validation after the common parts have been removed from the data-sets using an OPLS approach.

### Experimental

To test the three methods in different conditions we use simulated data. We will keep the model itself small with only 1 common component and 1 (or 2) individual component(s) per data-set. To generate the data we use the score and loading structure from Eqs. 2 and 3.

$$ \begin{array}{l}{\mathbf{X}}_1={\mathbf{T}}_c{\mathbf{P}}_{c_1}^{\mathrm{t}}+{\mathbf{T}}_{d_1}{\mathbf{P}}_{d_1}^{\mathrm{t}}+{\mathbf{E}}_1\hfill \\ {}{\mathbf{X}}_2={\mathbf{T}}_c{\mathbf{P}}_{c_2}^{\mathrm{t}}+{\mathbf{T}}_{d_2}{\mathbf{P}}_{d_2}^{\mathrm{t}}+{\mathbf{E}}_2\hfill \end{array} $$

The scores **T**_*c*_, ${\mathbf{T}}_{d_1}$ and ${\mathbf{T}}_{d_2}$ are drawn from a standard normal distribution in such a way that they are orthogonal to each other. Then each scores vector was scaled to length 1. The error terms **E**_1_ and **E**_2_ are based on pseudo numbers drawn from a standard normal distribution. The data-sets have 70 observations each (*I* = 70) and **X**_1_ contains 100 variables (*J*_1_ = 100) and **X**_2_ 50 variables (*J*_2_ = 50). The data of each data-set is column-centered and the variance of each block is scaled to unit variance. In our examples we have chosen a set of spectral loadings for illustrative purposes. In functional genomics data-sets e.g. transcriptomics or metabolomics data a similar situation can be envisioned when in functional groups the features are expected to highly correlate. The latent components then describe structured variation of the functional groups over the objects.

The three methods will be evaluated using the model settings that were suggested by the original model estimation procedure of each method respectively and if different from the actual model, with the real model settings as well. Two different scenarios are evaluated in which two different situations are simulated for the two data-sets:

1. Scenario 1, abundant variation in common loadings, almost orthogonal loadings
2. Scenario 2, low abundant variation in common loadings, almost orthogonal loadings

**Fig. 2:**
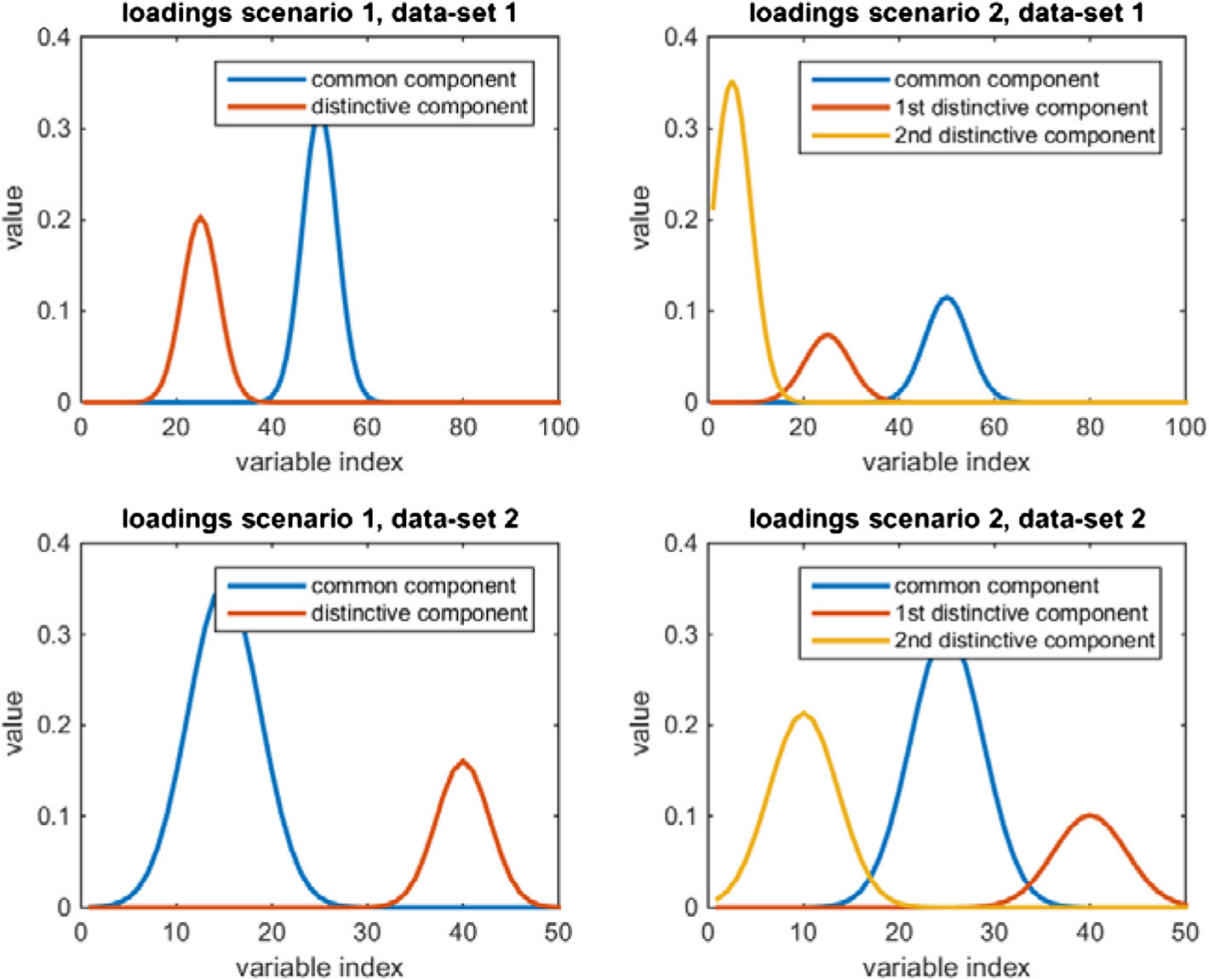
The loadings that were used to generate the data with for both scenarios

Figure 2 shows the loadings that are used to generate the data of the two blocks for both scenarios. The contributions of the distinctive and common parts for the different scenarios are listed in Additional file 1: Table S1 and Table 2 (Scenario 1: (0.66^c1^/0.28^d1^ and 0.85^c2^/0.13^d2^), scenario 2: (0.11^c1^/0.88^d1^ and 0.62^c2^/0.36^d2^)). The first scenario should give insight in the performance of the methods under conditions well suited to find the common variation. The second scenario should reveal issues for data that is more realistic like for example, the detection and removal of batch effects.

**Table 2:** Summary table of explained variances by the different methods in the second scenario using the real model settings (1,2,2)

Data-set	Part	Real	DISCO	JIVE	O2-PLS
1	Common	0.11	0.11	0.83	0.11
1	Distinctive	0.88	0.88	0.16	0.88
1	Error	0.01	0.01	0.01	0.01
2	Common	0.62	0.62	0.00	0.62
2	Distinctive	0.36	0.36	0.91	0.32
2	Error	0.02	0.02	0.09	0.06

The three methods will also be applied to experimental data from GlioBlastoma Multiform (GBM) brain tumors available at The Cancer Genome Atlas (TCGA). The mRNA (234 × 23293) and miRNA (234 × 534) data-sets describe the messenger RNA’s and small RNA’s profiles of 234 subjects that suffer from different kinds of brain tumors. The same data was already analysed by JIVE in its original paper [12]. Here we use it for comparison of JIVE and the other two methods.

## Results

### Scenario 1, abundant common variance, almost orthogonal loadings

The data sets in the 1^st^ scenario did not lead to any problems. All three methods properly select the model of common and distinctive components (i.e. 1 common, and 1 distinctive component for each data-set). The results of DISCO, JIVE and O2-PLS almost exactly match the simulated scores and loadings, which from a mathematical point of view is also expected (see Appendix, “Observations on JIVE, SCA and covariance”). The loadings are plotted in Additional file 1: Figure S3. The correlation of the fitted scores with the original scores is 1 for all methods.

Additional file 1: Table S1 summarizes the explained variances for the fitted results by the different models. The different methods decompose the two data-sets into the same common and distinctive parts. As discussed earlier, the errors for JIVE and O2-PLS are not orthogonal to the common parts and therefore cannot be calculated as the difference of **X**_***k***_ and the common and distinctive variance combined (**C**_*k*_ + **D**_*k*_). In this case however, the data was fabricated with orthogonal common and distinctive scores and we were able to calculated the error as the difference. Furthermore ∥**C**_*k*_**C**_*k*_^+^**E**_*k*_∥^2^ ≪ ∥**E**_*k*_∥^2^ which implies that the projection of **E**_*k*_ on **C**_*k*_ is very small indeed.

### Scenario 2, low abundant common variance, almost orthogonal loadings

In the second scenario the model was made more complex with less abundant common variance and more distinctive components per data-set. The difference between the methods already becomes apparent in the model selection. Additional file 1: Table S2 shows the estimated number of component models for the different methods. Each of the three methods selects a different ‘best’ model. With the O2-PLS cross-validation the ‘real’ model is selected. Both JIVE and DISCO select 0 common components.

**Fig. 3:**
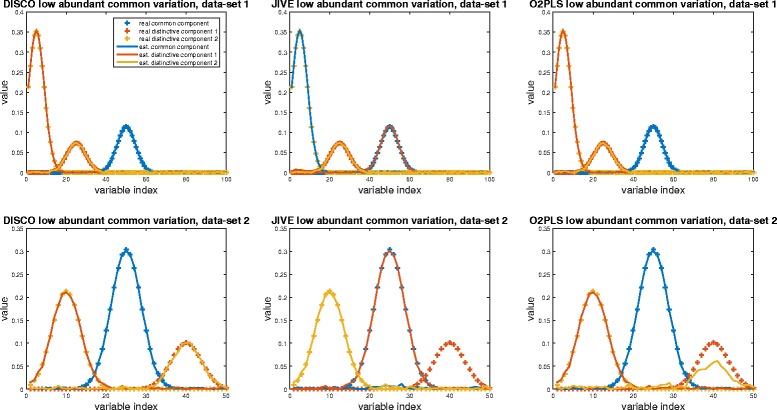
Real and fitted common and distinctive parts for each methods when there is an low abundant common variation (scenario 2). The real common component is show with a blue cross, the first and second distinctive component with an orange and yellow cross. The fits by the methods are identified by a line with in their respective color

For completeness, the loading plots and score assessments of the decompositions of JIVE and DISCO with the suggested model settings are included in the Additional file 1. The estimated common and distinctive loadings for the methods with the real model settings (1,2,2) are shown in Fig. 3.

The DISCO results with the ‘real’ model settings show a perfect decomposition in loadings and scores for both data-sets. The JIVE results show that all three components of the first data-set are fitted perfectly but that the common component is identified incorrectly; the component with the largest variance is identified as common. Because of the orthogonality restriction of **C**_1_**D**_1_^*t*^ = 0 and **C**_1_**D**_2_^*t*^ = 0., the real common component in data-set 2 cannot be selected anymore which results in a score vector of zero (the blue line). The two remaining distinctive components are used to fit the two loadings with the largest variation.

In JIVE the first step is to select the allocated number of common components. At this stage this selection is only determined by the largest variance, regardless whether or not this is ‘real’. If this selected part happens to be the distinctive part, the ‘real’ distinctive part is designated as common variance. In these cases the JIVE algorithm is not able to classify it as common, even after all the iterations. This behavior is investigated further by generating different data-sets with increasing variation in the common component. For each data-set the JIVE decomposition is run and the proper identification of the common and distinctive components is recorded (see Additional file 1). Only when the total common variation is larger than the variation of the largest distinctive component, the proper common component is identified.

The O2-PLS method suggested the real model complexity and the decomposition in loadings and scores show a good fit to the original data. The loading profiles show a good fit for the first data-set but for the second data-set the smallest individual component is under-estimated. This is also reflected in the amount of explained distinctive variation for the second data-set. Table 2 summarizes the explained variation for the fitted blocks by the different models. All methods steer towards a maximum amount of explained variation. Again, the residuals were determined as differences with the original data because the data was generated with orthogonal scores and ∥**C**_*k*_**C**_*k*_^+^**E**_*k*_∥^2^ ≪ ∥**E**_*k*_∥^2^.

### GlioBlastoma

The mRNA and miRNA measurements of Glioblastoma cells were used in the JIVE paper to introduce the method. We use the data to compare JIVE to DISCO and O2-PLS. We adopted the model settings that were found by the permutation approach (i.e. 5 common components, 33 distinctive components for mRNA and 13 for miRNA). For completeness the optimal number of components for the models was estimated again with each model selection method and the results are shown in Additional file 1: Table S4. The data were mean centered for each feature and each data-set was normalized to unit sum of squares. The data concerns different types of brain tumor cells.

**Fig. 4:**
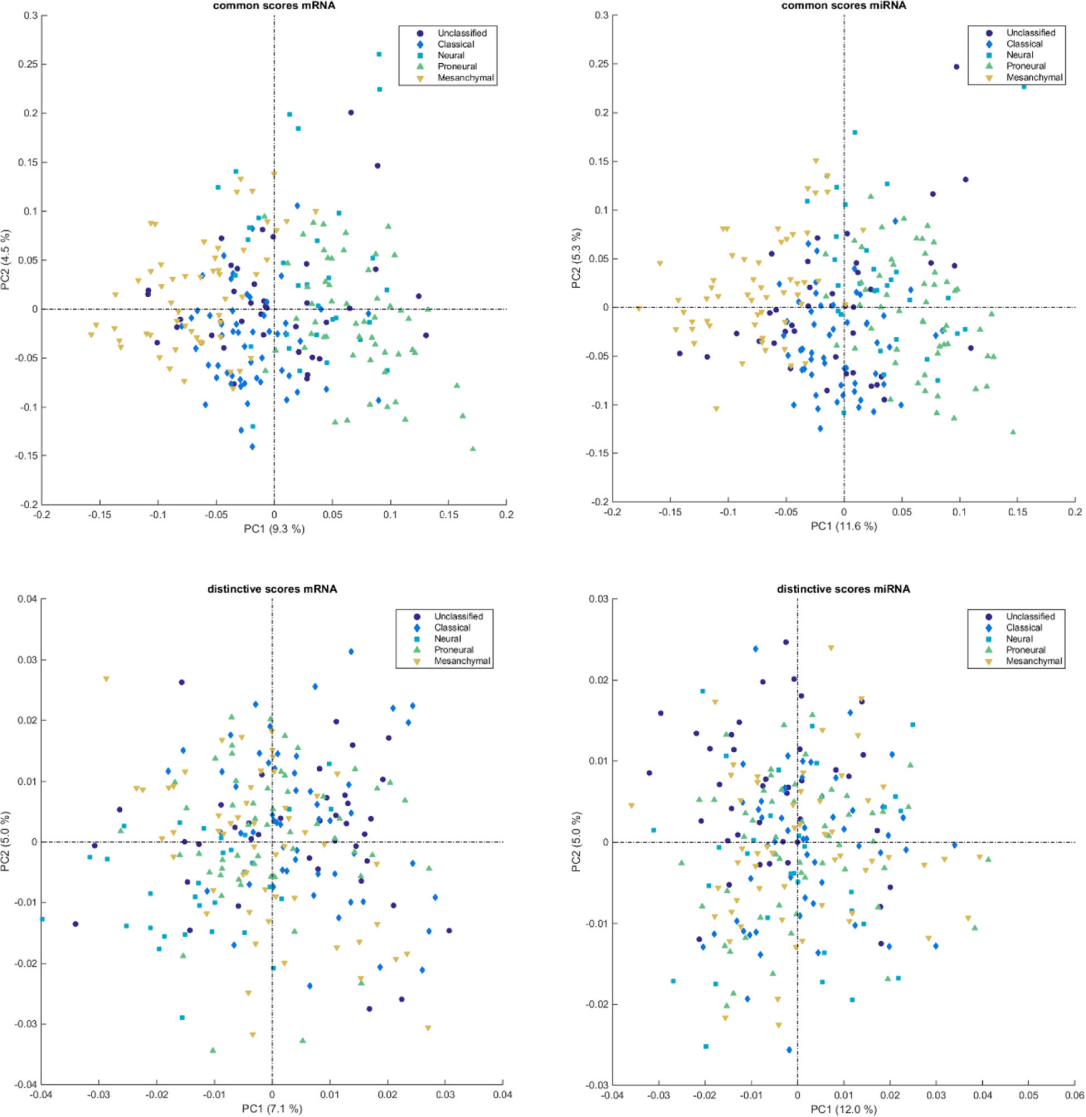
Score plots of the common (*top row*) and distinctive (*bottom row*) parts of the mRNA data-set (*left column*) and miRNA data-set (*right column*) after O2-PLS decomposition

As an example the O2-PLS score plots for both mRNA and miRNA for the common and distinctive parts are presented in Fig. 4. The common part shows a much clearer separation between the groups than the distinctive parts. The explained common and distinctive variation of the methods are listed in Table 3. With the exact same model settings, the JIVE method is able to explain approximately 5 % more of combined distinctive and common variation than DISCO and O2-PLS (∥**C**_*k*_**C**_*k*_^+^**E**_*k*_∥^2^ ≪ ∥**E**_*k*_∥^2^ for both data-sets). In comparison to DISCO and O2-PLS, JIVE describes less common variation but more distinctive variation. This phenomenon can possibly be accounted for by the iterative behavior of JIVE. By iteratively estimating the common and distinctive parts from only a selected part of the variation in the data, the common part seems less affected by over fitting. This phenomenon is further discussed in the Additional file 1.

**Table 3:** Summary table of fitted explained variation by the different methods using the real mRNA and miRNA data-sets

Data-set	Part	DISCO	JIVE	O2-PLS
mRNA	Common	0.22	0.15	0.20
mRNA	Distinctive	0.45	0.57	0.48
mRNA	Total	0.68	0.72	0.68
miRNA	Common	0.33	0.26	0.29
miRNA	Distinctive	0.40	0.49	0.40
miRNA	Total	0.73	0.75	0.70

**Fig. 5:**
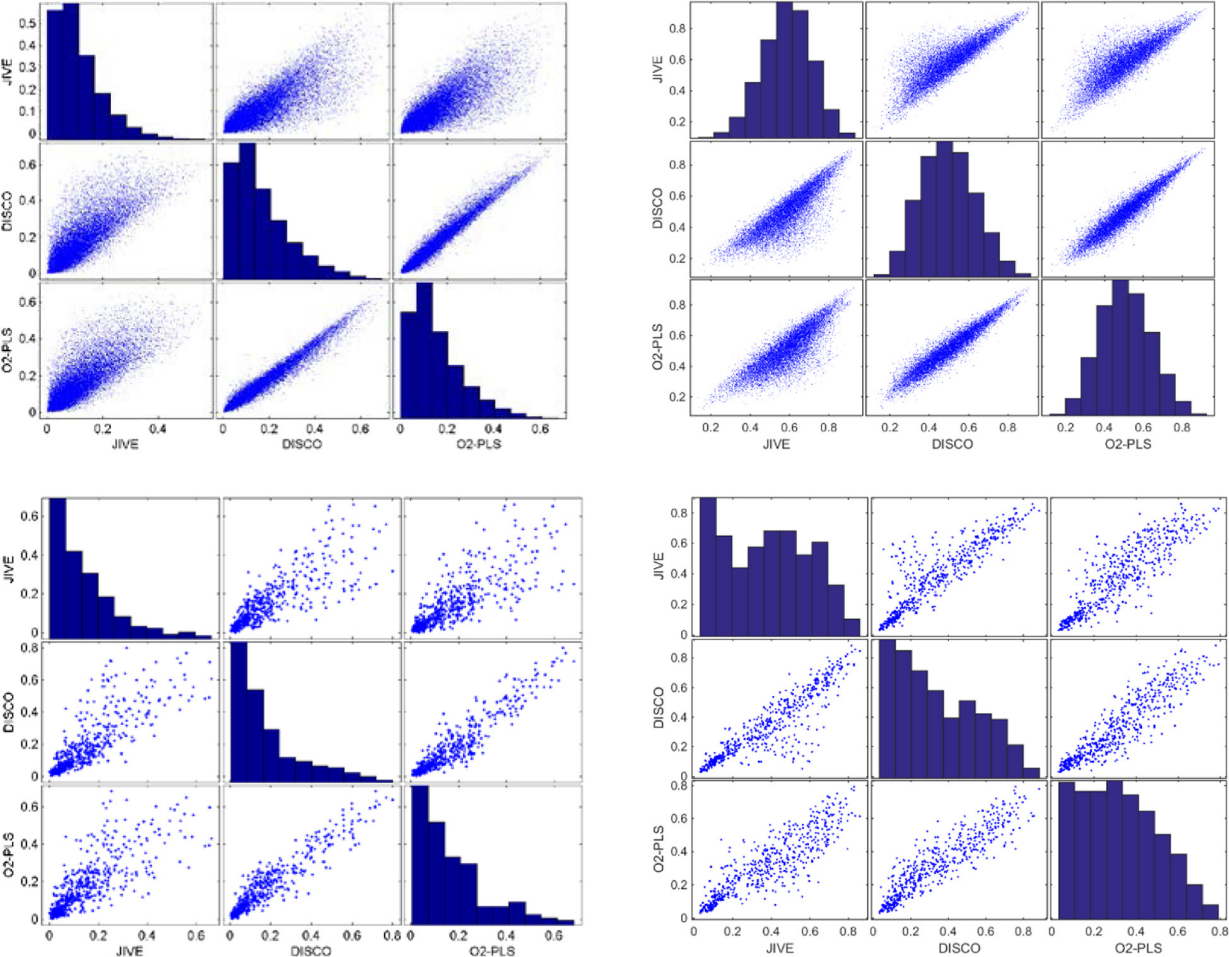
Scatter plots of % variance of original variable explained in common parts (*left*) and % variance of original variable explained in distinctive parts (*right*) of the mRNA data-set on the top row and of the miRNA on the bottom row between the the different algorithms. DISCO and O2-PLS look very similar. JIVE shows more genes of which more variance is used in the distinctive part which coincides with the increased amount of distinct variance explained by JIVE in comparison to DISCO and O2-PLS

To study the overlap in the three methods, the percentages explained variation in the common part and in the distinctive part per gene are plotted against each other in Fig. 5 for mRNA and miRNA. On the left side the results of the common parts are given. The explained variation for the genes in the common part using O2-PLS and DISCO are strongly correlating. The explained variation for the genes using JIVE is clearly different. The common part in JIVE describes a lower amount of explained variation than the other methods. The distinctive part (on the right-hand side) shows the same phenomenon. Again the explained variation for the distinctive part is similar using O2-PLS and DISCO, while JIVE now describes a higher amount of explained variation. The figures on the diagonal show the distribution in explained variation for each of the 3 methods. This is very similar for the three methods. What is striking however is the difference in distribution of explained variation between the common and distinctive parts. In the common part, most genes are hardly explained while a low number of genes is highly explained. For the distinctive part no such preference is observed and a normal like distribution of explained variation is obtained.

For the miRNA, the situation is similar to the mRNA data. Again JIVE has a lower explained variation for each miRNA in the common part and a higher explained variation in the distinctive part compared to DISCO and O2-PLS. The distribution of the explained variation of the distinctive part is clearly different than for the mRNA. For miRNA, still many features are not well described. This could be related to a lower amount of systematic variation in the miRNA’s and consequently, lower correlation between the different miRNA’s. Therefore, each component only describes few miRNA features.

One explanation for DISCO is that orthogonality restrictions prohibit optimal fitting and as a result the cross over variation (i.e. the variation for miRNA explained by the distinctive score for the mRNA) is significant. For miRNA this was 13 % and for mRNA this was 4 % of the total variance. This amount of cross over variation is much larger for miRNA than mRNA because the 33 distinctive mRNA components all add to the cross over variation of miRNA compared to only 13 components vice versa.

In the O2-PLS method the initial common scores (${\mathbf{T}}_{c_k}$) are estimated from the initial loadings (**P**_k_) and original data (**X**_*k*_). The distinctive components are removed from the remainder ${\mathbf{R}}_k\ \left({\mathbf{R}}_k={\mathbf{X}}_k-{\mathbf{T}}_{c_k}{\mathbf{P}}_{c_k}^{\mathrm{t}}\right)$ and **X**_*k*_ is updated. However, in the final step (step 12 in the O2-PLS algorithm see Appendix), the common part is recalculated from the updated **X**_*k*_. This recalculation gives a lower amount of variation for the common part than before **X**_*k*_ was deflated with distinct components. This variation can neither be described by the distinctive nor common part of the model anymore. Large discrepancies indicate that the estimation of the initial common part contained larger amounts of orthogonal variation. After ${\mathbf{T}}_{c_k}$ has been re-estimated, the distinctive part is not recalculated anymore. Perhaps more total variance could have been accounted for if O2-PLS would have used an iterative procedure like JIVE, which is fully iterative.

The score plots of the common and distinctive parts for the different methods all reveal a better separation of the classes in the common part of the miRNA data-set. To indicate the quality of class separation we adopted the standardized subtype within sums of squares (SWISS) from the original JIVE paper. This represents the variability within subtypes (across all rows) as a proportion of total variability. A lower score indicates better class separation. Table 4 shows the SWISS scores for both data sets using all three methods. The SWISS score for the common parts is compared to the SWISS scores of a 5 component PCA solution of both sets to see whether the removal of the distinctive information would provide a better set of common scores compared to the normal PCA scores. For O2-PLS we see a slight improvement to a SWISS of 0.65, while the JIVE SWISS score is worse (0.74). We see that the distinctive parts of the data have lost their discriminative power. Note that the SWISS for the common parts for both data sets is exactly the same for DISCO and JIVE as the common scores are the same for those methods.

**Table 4:** Summary of the SWISS scores for the common and distinctive parts identified by the different models during the analysis of the mRNA/miRNA GlioBlastoma data

	Common (mRNA/miRNA)	Distinctive mRNA	Distinctive miRNA
REAL (5 PC’s)	0.66/0.79		
DISCO	0.67	0.94	0.99
JIVE	0.74	0.92	0.94
O2-PLS	0.65/0.66	0.97	0.93

The high correspondence in explained variation for each mRNA and miRNA feature between DISCO and O2-PLS is corroborated by their scores. Table 5 shows the RV matrix correlation [25] between the scores of the different methods. Again a high correlation between the O2-PLS and DISCO scores are observed for the common part. For the distinctive part this cannot be observed.

**Table 5:** RV modified coefficients of the common and distinctive scores for GlioBlastoma data-sets

Data-set	Part	Method	O2-PLS	DISCO	JIVE
mRNA	Common	O2-PLS	X	0.77/0.67	0.42/0.41
mRNA	Common	DISCO	0.77/0.67	X	0.58
mRNA	Common	JIVE	0.42/0.41	0.58	X
mRNA	Distinctive	O2-PLS	X	0.53	0.58
mRNA	Distinctive	DISCO	0.53	X	0.68
mRNA	Distinctive	JIVE	0.58	0.68	X
miRNA	Distinctive	O2-PLS	X	0.56	0.55
miRNA	Distinctive	DISCO	0.56	X	0.74
miRNA	Distinctive	JIVE	0.55	0.74	X

## Discussion and conlusions

The three methods discussed in this paper to separate common from distinct information all use different approaches, which lead to slightly different models of the data. What is exactly common variation and what is distinctive depends on the different orthogonality constraints applied and the algorithms used to estimate these different parts. When the common variation is abundant, all methods are able to find the correct solution. With real data however, complexities in the data are treated differently by the three methods.

Due to fewer orthogonality constraints that are imposed by JIVE and O2-PLS, there is more freedom to select the scores and loadings for the two data-sets. This freedom is not present in DISCO which has the most severe orthogonality restrictions. In the two scenarios shown in this paper, all scores and loadings were chosen orthogonal. Therefore DISCO was able to find the correct scores and loadings while JIVE and O2-PLS found variations thereof that still obayed their orthogonality assumptions. In case of less abundant common variation, both JIVE and DISCO failed to detect the proper amount of common components which can be understood from the methods themselves. Not knowing the real model however can give rise to unexpected results while decomposing the data in common and distinctive components.

Even with the optimal model settings selected the JIVE method is the most susceptible to identifying the wrong common components. Due to the SCA of the concatenated matrix JIVE has problems finding common components especially when they are smaller than a distinctive component in one of the blocks. If the common and distinctive variation is approximately of the same magnitude, JIVE is able to properly identify them due to its iterative nature. JIVE re-estimates the common and distinctive parts until they converge, while O2-PLS, which only once re-estimates the common part once, seems to be stuck in a sub optimal solution for the distinctive part.

When small data sets with a low number of features (J_k_ < I) are used, these data sets may not be well represented by the common scores in JIVE, and even worse, the common scores present information that is not even present in these blocks. This may lead to misinterpretation of both common scores and distinctive scores of such a block [24]. The O2-PLS algorithm is the most flexible one and allows the separate and distinctive parts to be determined using block scores instead of super scores. This way no information is transferred from one data-set to the other. The distinctive parts however, are also limited by orthogonality constraints and therefore have a biased interpretability.

In the real data example the three methods all selected a smaller number of common than distinct components. In contrast to the simulations, O2-PLS suggested a smaller number of common components than JIVE and DISCO. This could possibly indicate an over estimation of the number of common components by DISCO and JIVE. It was shown that the lack of structure in the raw miRNA data-set has been replaced by an apparent structure in the common part. The combination of the data-sets has revealed a subset of miRNA’s that mathematically can be linked to the mRNA’s by all three methods. Because the methods are not supervised, the appearing structure gives rise to further biological interpretation of not only the common parts but also the distinctive parts. In situations like these, DISCO, JIVE and O2-PLS can be considered to act as pre-processing steps (i.e. filtering steps).

In summary, all three methods have their own approach to estimate common and distinctive variation with their specific strength and weaknesses. Due to their orthogonality properties and their used algorithms their view on the data is slightly different. By assuming orthogonality between common and distinctive, true natural or biological phenomena that may not be orthogonal at all might be misinterpreted.

## Appendix

### List of used symbols

**Table:**

*K*	total number of datasets, *k* = 1.. *K*
*I*	number of rows (objects)
*J*_*k*_	number of columns (variables) for matrix *k*
*J*	total number of variables (∑_1_^*K*^ *J*_*k*_)
*c*_*c*_	number of components for common part
*c*_*k*_	number of components for distinctive part of matrix *k*
*c*_*t*_	total number of components ((∑_*k* = 1_^*K*^ *c*_*k*_) + *c*_*c*_)
**X**_*k*_	data matrix (*I* × *J*_*k*_)
**X**	concatenated data matrix [**X**_*k*_| … |**X**_*K*_](*I* × *J*)
**C**_*k*_	common part of matrix *k* (*I* × *J*_*k*_)
**C**	concatenated common parts [**C**_*k*_| … |**C**_*K*_](*I* × *J*)
**D**_*k*_	distinctive part of matrix *k* (*I* × *J*_*k*_)
**D**	concatenated distinctive parts [**D**_*k*_| … |**D**_*K*_] (*I* × *J*)
**E**_*k*_	the residual error of matrix *k* (*I* × *J*_*k*_)
**E**	concatenated residual errors [**E**_*k*_| … |**E**_*K*_] (*I* × *J*)
**T**_*sca*_	scores of SCA model (corresponds to objects) (*I* × *J*_*t*_)
**P**_*sca*_	loadings of SCA model (corresponds to variables) (*J* × *c*_*t*_)
**P***	rotation target loading in DISCO model (*J* × *c*_*t*_)
**B**	rotation matrix in DISCO (*c*_*t*_ × *c*_*t*_)
**W**	weight matrix (used in DISCO) to penalize rotation matrix (*J* × *c*_*t*_)
**T**_*c*_	common scores (SCA and JIVE) (*I* × *c*_*c*_)
**P**_*c*_	common loadings (JIVE) (*I* × *c*_*c*_)
${\mathbf{T}}_{c_k}$	common scores (O2-PLS) for matrix *k* (*I* × *c*_*c*_)
${\mathbf{P}}_{c_k}$	common loadings for matrix *k* (*J*_*k*_ × *c*_*c*_)
${\mathbf{T}}_{d_k}$	distinctive scores for matrix *k* (*I* × *c*_*k*_)
${\mathbf{P}}_{d_k}$	distinctive loadings for matrix *k* (*J*_*k*_ × *c*_*k*_)
∘	Hadamard (element-wise) matrix product

### Algorithms

#### DISCO

1. Define a target loading matrix (**P***) of zeros and ones based on the model that was defined by the common and distinctive components (*c*_*c*_, *c*_1_, and *c*_2_);
  $$ {\mathbf{P}}^{*}=\left[\begin{array}{lll}{1}^{J_1\times {c}_1}\hfill & {0}^{J_1\times {c}_2}\hfill & {1}^{J_1\times {c}_c}\hfill \\ {}{0}^{J_2\times {c}_1}\hfill & {1}^{J_2\times {c}_2}\hfill & {1}^{J_2\times {c}_c}\hfill \end{array}\right] $$
2. Define the weight matrix as **W** = 1 − **P***, where **1** is a matrix of ones.
3. **X** = [**X**_1_|**X**_2_]
4. $$ \mathbf{X}={\mathbf{U}}_{\left({\boldsymbol{c}}_{\boldsymbol{t}}\right)}{\mathbf{S}}_{\left({\boldsymbol{c}}_{\boldsymbol{t}}\right)}{V}_{\left({\boldsymbol{c}}_{\boldsymbol{t}}\right)}^t $$
5. $$ {\mathbf{T}}_{sca}={\mathbf{U}}_{\left({\boldsymbol{c}}_{\boldsymbol{t}}\right)} $$
6. $$ {\mathbf{P}}_{sca}={\mathbf{V}}_{\left({\boldsymbol{c}}_{\boldsymbol{t}}\right)}{\mathbf{S}}_{\left({\boldsymbol{c}}_{\boldsymbol{t}}\right)} $$
7. Randomly initialize **B**^0^ subject to **B**^0t^**B**^0^ = **I** = **B**^0^**B**^0t^
8. Intialize iteration index *l* = 1
9. **Y** = **P**_*sca*_**B**^*l* − 1^ + **W** ∘ (**P*** − **P**_*sca*_**B**^*l* − 1^)
10. [**U**_*r*_, **S**_*r*_, **V**_*r*_] = *svd*(**Y**^t^**P**_*sca*_)
11. **B**^*l*^ = **V**_*r*_**U**_*r*_^t^
12. Compute *h*(**B**^*l*^) = ∥ **W** ∘ (**P**_*sca*_**B**^*l*^ − **P***) ∥ ^2^
13. Repeat step **9-12** until *h*(**B**^*l*^) − *h*(**B**^*l* − 1^) < *τ* or *l* > *l*_*max*_

After convergence and because **B** is subject to **B**^t^**B** = **I** the rotated scores and loadings can be calculated via **T**_*r*_ = **T**_**sca**_**B** and **P**_*r*_ = **P**_**sca**_**B**. The common and individual scores (**T**_*c*_ and ${\mathbf{T}}_{d_i}$) and loadings (${\mathbf{P}}_{c_i}$ and ${\mathbf{P}}_{d_i}$) are separated according to the target matrix (**P***). The loadings can then be determined as specific subsets of the rotated loadings to calculate the terms from Eq. 1:

$$ {\mathbf{C}}_1={\mathbf{T}}_c{\mathbf{P}}_{c_1}^{\mathrm{t}},\ {\mathbf{C}}_2={\mathbf{T}}_c{\mathbf{P}}_{c_2}^{\mathrm{t}},\ {\mathbf{D}}_1={\mathbf{T}}_{d_1}{\mathbf{P}}_{d_1}^{\mathrm{t}}\mathrm{and}\ {\mathbf{D}}_2={\mathbf{T}}_{d_2}{\mathbf{P}}_{d_2}^{\mathrm{t}} $$

#### JIVE

1. **X** = [**X**_1_|**X**_2_]
2. $$ \mathbf{X}={\mathbf{U}}_{\left({c}_c\right)}{\mathbf{S}}_{\left({c}_c\right)}{\mathbf{V}}_{\left({c}_c\right)}^t $$
3. **P**_*c*_ = **V**_*c*_**S**_*c*_
4. **C** = **T**_***c***_**P**_***c***_^**t**^
5. **R**_***k***_ = **X**_*k*_ − **C**_*k*_
6. $$ {\mathbf{R}}_k-{\mathbf{T}}_{c_k}{\mathbf{T}}_{c_k}^{\mathbf{t}}{\mathbf{R}}_k={\mathbf{U}}_{d_k\left({c}_k\right)}{\mathbf{S}}_{{\boldsymbol{d}}_{\boldsymbol{k}}\left({c}_k\right)}{\mathbf{V}}_{d_k\left({c}_k\right)}^{\boldsymbol{t}} $$
7. $$ {\mathbf{T}}_{d_k}={\mathbf{U}}_{d_k} $$
8. $$ {\mathbf{P}}_{d_k}={\mathbf{V}}_{d_k}{\mathbf{S}}_{d_k} $$
9. $$ {\mathbf{D}}_k={\mathbf{T}}_{d_k}{\mathbf{P}}_{d_k}^{\mathrm{t}} $$
10. **X** = **X** − [**D**_1_|**D**_2_]
11. Repeat steps **2** through **11** until convergence of **C** + **D**, where **C** = [**C**_1_|**C**_2_] and **D** = [**D**_1_|**D**_2_]

#### O2-PLS

Slightly different implementations were published which leaves room for possible different interpretations. In our implementation we followed the pseudo code described by Löfstedt et al ([20, 26, 27]) and made sure that our O2-PLS results corresponded to the 2 data-set OnPLS results.

1. $$ {\mathbf{X}}_2^{\mathrm{t}}{\boldsymbol{X}}_1={\mathbf{P}}_{c_1\left({c}_{\mathrm{c}}\right)}{\mathbf{D}}_{\left({c}_{\mathrm{c}}\right)}{\mathbf{P}}_{c_2\left({c}_{\mathrm{c}}\right)}^{\boldsymbol{t}} $$
2. Intialize iteration index *l* = 1
3. $$ {\mathbf{T}}_{c_k}={\mathbf{X}}_k{\mathbf{P}}_{\mathrm{k}} $$
4. $$ {\mathbf{R}}_k={\mathbf{X}}_k-{\mathbf{T}}_{c_k}{\mathbf{P}}_{c_k}^{\mathrm{t}} $$
5. $$ {\mathbf{R}}_k^{\mathrm{t}}{\mathbf{T}}_{c_k} = {\mathbf{u}}_{d_k(1)}{\mathbf{s}}_{{\boldsymbol{d}}_{\boldsymbol{k}}(1)}{\mathbf{v}}_{d_k(1)}^{\boldsymbol{t}} $$
6. $$ {\mathbf{t}}_{d_{k,l}}={\mathbf{X}}_k{\mathbf{u}}_{d_k(1)} $$
7. $$ {\mathbf{p}}_{d_{k,l}}={\left({\mathbf{t}}_{d_{k,l}}^{\mathrm{t}}{\mathbf{t}}_{d_{k,l}}\right)}^{-1}{{\mathbf{X}}_k}^{\mathrm{t}}{\mathbf{t}}_{d_{k,l}} $$
8. $$ {\mathbf{X}}_k={\mathbf{X}}_k-{\mathbf{t}}_{d_{k,l}}{\mathbf{p}}_{d_{k,l}}^{\mathrm{t}} $$
9. Repeat steps 3 through 8 for the number of distinctive components per data-set (*l* = 1.. *c*_*k*_) and both data-sets (*k* = 1..2).
10. $$ {\mathbf{T}}_{d_k}=\left[{\mathbf{t}}_{d_{k,1}}\left|{\mathbf{t}}_{d_{k,2}}\right|\cdots \Big|{\mathbf{t}}_{d_{k,{c}_k}}\right] $$
11. $$ {\mathbf{P}}_{d_k}=\left[{\mathbf{p}}_{d_{k,1}}\left|{\mathbf{p}}_{d_{k,2}}\right|\cdots \Big|{\mathbf{p}}_{d_{k,{c}_k}}\right] $$
12. $$ {\mathbf{T}}_{c_k}={\mathbf{X}}_k{\mathbf{P}}_{c_k} $$

After these steps have been performed the elements from Eq. 1 can be calculated via

9 $$ {\mathbf{C}}_1={\mathbf{T}}_{c_1}{\mathbf{P}}_{c_1}^{\mathrm{t}},\ {\mathbf{C}}_2={\mathbf{T}}_{c_2}{\mathbf{P}}_{{\boldsymbol{c}}_2}^{\mathrm{t}},\ {\mathbf{D}}_1={\mathbf{T}}_{d_1}{\mathbf{P}}_{{\boldsymbol{d}}_1}^{\mathrm{t}}\ \mathrm{and}\ {\mathbf{D}}_2={\mathbf{T}}_{d_2}{\mathbf{P}}_{d_2}^{\mathrm{t}} $$

#### Observations on JIVE, SCA and covariance

If there is no distinctive information SCA and O2-PLS give the same result:

1. **X**_1_ = **T**_*c*_**P**_1_^***t***^ + **E**_1_ and **X**_2_ = **T**_*c*_**P**_2_^***t***^ + **E**_2_
  Without noise these equations reduce to:
2. **X**_1_ = **T**_*c*_**P**_1_^***t***^ and **X**_2_ = **T**_*c*_**P**_2_^***t***^
3. **X**_2_^***t***^**X**_1_ = (**T**_*c*_**P**_2_^***t***^)^***t***^(**T**_*c*_**P**_1_^***t***^) = **P**_2_(**T**_***c***_^***t***^**T**_*c*_)**P**_1_^***t***^
  **T**_*c*_ can be chosen such that **T**_***c***_^***t***^**T**_*c*_ = **I** so consequently:
4. **X**_2_^***t***^**X**_1_ = **P**_2_**P**_1_^***t***^

The analysis of the covariance matrix therefor will generate the same result if and only if there is no distinctive variation. This principle likely can be extended (no proof given) to those cases where the common variation is larger than the distinctive variation.

If there is a distinctive part it can be shown that an svd on the covariance matrix is less susceptible to larger distinctive parts and will better identify the common variation than the SCA approach used in JIVE.

1. **X**_1_ = **C**_1_ + **D**_1_ and **X**_2_ = **C**_2_ + **D**_2_
2. **X**_1_^***t***^**X**_2_ = (**C**_1_ + **D**_1_)^*t*^(**C**_2_ + **D**_2_) = **C**_1_^***t***^**C**_2_ + **D**_1_^***t***^**D**_2_

Because the common variation is expected to occur in both data-sets their cross product is expected to be larger than the crosspoduct of the distinctive parts that by definition should show less correlation between the subjects in both data-sets. The crossproducts of the distinctive and common parts can be neglected in comparison because of the orthogonality constraints (in O2-PLS).

## Additional file

Additional file 1: Supplementary data. This file contains supplementary Tables S1-S4 and Figures S1-S7. (DOCX 735 kb)
